# ThSSim: A novel tool for simulation of reservoir thermal stratification

**DOI:** 10.1038/s41598-019-54433-2

**Published:** 2019-12-06

**Authors:** Roohollah Noori, Fuqiang Tian, Guangheng Ni, Rabin Bhattarai, Farhad Hooshyaripor, Bjön Klöve

**Affiliations:** 1 School of Environment, College of Engineering, University of Tehran, Tehran, 1417853111 Iran; 2 Department of Hydraulic Engineering, State Key Laboratory of Hydroscience and Engineering, Tsinghua University, Beijing, 100084 China; 3 Department of Agricultural and Biological Engineering, University of Illinois at Urbana Champaign, 1304W Pennsylvania Ave, Urbana, IL 61801 USA; 4 Department of Civil Engineering, Science and Research Branch, Islamic Azad University, Tehran, 1477893855 Iran; 5 Water, Energy and Environmental Engineering Research Unit, University of Oulu, PO Box 4300, 90014, Finland, Oulu, Finland

**Keywords:** Hydrology, Limnology

## Abstract

This study presents a novel tool, ThSSim, for simulation of thermal stratification (ThS) in reservoirs. ThSSim is a simple and flexible reduced-order model-based the basis function (RMBF) that combines CE-QUAL-W2 (W2) and proper orthogonal decomposition (POD). In a case study, it was used to simulate water temperature in the Karkheh Reservoir (KR), Iran, for the period 2019–2035. ThSSim consists of two space- and time-dependent components that add predictive ability to the RMBF, a major refinement that extends its practical applications. Water temperature simulations by the W2 model at three-hour time intervals for the KR were used as input data to the POD model to develop ThSSim. To add predictive ability to ThSSim and considering that space-dependent components are not a function of time, we extrapolated the first three time-dependent components by September 30, 2035. We checked the predictive ability of ThSSim against water temperature profiles measured during eight sampling campaigns. We then applied ThSSim to simulate water temperature in the KR for 2019–2035. Simulated water temperature values matched well those measured and obtained by W2. ThSSim results showed an increasing trend for surface water temperature during the simulation period, with a reverse trend observed for water temperature in the bottom layers for three seasons (spring, summer and autumn). The results also indicated decreasing and increasing trends in onset and breakdown of thermal stability, respectively, so that the duration of ThS increased from 278 days in 2019 to 293 days in 2035. ThSSim is thus useful for reservoir temperature simulations. Moreover, the approach used to develop ThSSim is widely applicable to other fields of science and engineering.

## Introduction

Thermal stratification (ThS) is an important property of reservoirs and lakes that influences mixing regime and the physiochemical and biological characteristics of reservoirs^1^. Stratification is governed by a variety of driving forces including: long-wave radiation, sensible heat exchange, solar radiation, heat flux linked to evaporation and precipitation, outflow and inflow, wind, lake depth, thermal contact with the bottom of the water body, and light penetration through the water column^2–9^. These factors are mostly atmospheric forces, which usually oscillate over the year. In most climate zones, reservoir surface layers show a distinct temperature cycle during the year^5^, where these factors contribute to increasing and decreasing trends in water temperature in warm (summer) and cold (winter) seasons, respectively. This enhances ThS in reservoirs by establishment of a considerable temperature gradient between warm water in surface strata and cold water located in the bottom layers, and divides the water column into three distinct layers: epilimnion, metalimnion, and hypolimnion. The temperature gradient in the metalimnion acts as a barrier between epilimnion and hypolimnion, so that the latter gradually becomes anoxic and rich in nutrients due to mineralization of phytoplankton and diffusion of nutrients from bottom sediments. Meanwhile, the epilimnion becomes rich in dissolved oxygen (DO) due to dioxygen diffusion from the atmosphere and poor in nutrients due to uptake of nutrients by phytoplankton that largely sink down to the bottom^10,11^. The anoxia in the hypolimnion causes water quality deterioration due to diffusion of trouble-causing pollutants from the bottom sediments^12–14^. This ecological consequence is more important for deep monomictic lakes affected by ThS for most of the year, so that they only experience an overturn in winter.

Considering the above, understanding thermal stratification is important when seeking to understand and predict reservoir water quality. Numerical hydrodynamic and water quality models have been widely used for water quality predictions, because they appropriately consider the simultaneous effects of different driving forces on water temperature variations along the water column in reservoirs^15–22^. Most numerical models are able to reproduce water temperature in reservoirs, but modeling vertical and longitudinal directions is time-consuming if all driving forces on ThS are considered. In addition, large volumes of model outputs may need to be post-processed by a separate program, to capture the dominant modes of ThS during the simulation period or scenarios. To appropriately address these issues in modeling, the reduced order modeling approach based on proper orthogonal decomposition (POD) has been introduced in the field of computational fluid dynamics, with the aim of shortening the computing time and conserving model accuracy^23^. While this approach has been widely applied in the field of fluids engineering^23–27^, only a few studies have applied it in the field of ThS and water quality modeling. In one such study, nitrate and temperature variations in a large, deep reservoir were simulated with a reduced-order model-based basis function (RMBF) developed by linking the CE-QUAL-W2 (W2) model with POD^28,29^. In another, Kheirabadi *et al*.^30^ simulated surface currents in the Goragan Bay using a RMBF based on the MIKE3-FM model and POD. However, Noori *et al*.^29^ concluded that these RMBFs simply regenerate the results during the simulation period and are not well organized to predict targets in the future. Therefore, more studies are needed to extend the capability of RMBF in the field of hydrodynamics and water quality modeling, to identify limitations, and to improve model performance.

In this study, we developed and tested a tool for simulation of ThS in reservoirs. This tool, which we call ThSSim, is a RMBF consisting of W2 and POD models for simulation of ThS in a deep monomictic reservoir and for predicting it in the future. It therefore represents an important addition to the RMBFs developed by Noori *et al*.^28,29^ and Kheirabadi *et al*.^30^. In a wide perspective, ThSSim adds predictive ability to the RMBF.

## Materials and Methods

### Study area

The Karkheh Dam, the largest embankment dam in the Middle East, is built on the Karkheh River, which has the third highest river flow rate in Iran (Fig. S1). This river supplies water to more than four million people and is the main source of water for the Karkheh Reservoir (KR). The reservoir was built in 2001 to generate hydropower, irrigate downstream agricultural land, and control devastating flooding by the Karkheh River. The reservoir is 64 km long, covers an area of about 160 km^2^, and has a capacity at operating level of around 5.9 billion m^3^. It is the most important component of a network of reservoirs in western Iran designed to protect people from flooding and provide water for different purposes (Fig. S2). The KR is a deep monomictic lake with a maximum depth of 120 m at operating level that stratifies in most months of the year and only experiences mixing during winter. The large volume of the hypolimnion in this deep reservoir makes it difficult for the water column to be fully overturned during mixing^31^, so the mixing process in the KR is short-lived.

The Karkheh River Watershed (KRW) is around 43,000 km^2^ in area and consists of mountainous areas in the north and plains and foothills in the south. Therefore, the KRW has a wide range of weather conditions, with the mountainous regions in the north having cold winters and mild summers and the plains in the south being semi-arid with mild winters and long, hot summers. Mean annual rainfall in the reservoir area is 290 mm, mean annual temperature is 25 °C, and mean annual free water surface evaporation is around 2080 mm. The minimum and maximum recorded temperature during the year is −4.2 and 53.6 °C, respectively, and, on average, there are 4.5 freezing days per year and an annual average of 2763 sun-hours at the reservoir site. Mean and maximum recorded wind speed is 2.5 and 41 m/s, respectively, and the wind usually blows from the west. Based on long-term historical data (50-year period) measured at the Pay-e Pol gauging station, mean annual inflow into the KR is around 5.9 billion m3. According to Iran Water & Power Resources Development Company (IWPCO), four months (February to May) contribute more than 60% of inflow to the KR, with the maximum inflow usually occurring in April^32^.

### Thssim model development

Model development consisted of four phases: “W2 Model Application”, “ThSSim Model Development”, “ThSSim Model Validation”, and “ThSSim Model Application” (see Fig. S3). Specifically, ThSSim is an enhancement of the model developed by Noori *et al*.^31^ that links W2 and POD for regeneration of water temperature in the KR. Those authors developed a RMBF that accurately regenerates the water temperature in the KR by dividing the reservoir into 64 1-km long segments and 117 1-m vertical layers so that the computational domain comprises 1866 cells. They then calibrated and verified W2 using 15-month historical data. Noted that the air temperature, dew point temperature, wind, and cloud cover were used as atmospheric forcing data to calculate the heat budget of W2. The simulated water temperature in the KR was extracted at three-hour intervals in all grids to prepare data for POD application. In the present study, we used the calibrated and verified W2 model described by Noori *et al*.^31^ to calculate water temperature in the KR from October 1, 2005 to September 30, 2014, as shown in the subdivision “W2 Model Application” in Fig. S3. We then used the simulated water temperature data as input for development of ThSSim. Considering the discretization, the spatial resolution of the ThSSim was set at 1000 m in length and 1 m in depth, while the temporal resolution was three hours. Therefore, the desired resolution had to be defined for ThSSim in this step, although it is possible to develop it with finer/coarser grids and shorter/longer time intervals. However, selection of very fine grids and very short time intervals will increase the computing costs of ThSSim, while not significantly improving the results.

To develop ThSSim, as shown in the subdivision “ThSSim Model Development” in Fig. S3, three important features of RMBF were considered:

I. RMBF works well for periodic phenomena^33^. Water temperature variation in reservoirs is also periodic, since it fluctuates annually as a function of atmospheric driving forces and follows a clear trend during the year.
II. A target variable is decomposed into modes consisting of two space- and time-dependent components in RMBF^23^. This means that the space-dependent components are a function of **x** (**Ω**(**x**)), so that they do not change over time, while the time-dependent components are only a function of time (*τ*(*t*)) and change during year. This can be expressed as follows:
III. The ensemble of instantaneous space water temperature data simulated by W2 model at different time steps, called snapshots, consists of the set:

1 $$\delta =\{{{\bf{T}}}^{(i)}:\,1 < i < N\}$$

where **T** is the matrix of snapshots and *N* is the number of snapshots.

Statistically, the variable **T** is expressed as:

2 $${\bf{T}}({\bf{x}},t)=\bar{{\bf{T}}}({\bf{x}})+\hat{{\bf{T}}}({\bf{x}},t)\,{\rm{where}}\,\bar{{\bf{T}}}({\bf{x}})=\frac{1}{N}{\sum }_{i=1}^{N}{\bf{T}}({\bf{x}},t)$$

where *t* is time, $\bar{{\bf{T}}}({\bf{x}})$ is **T** averaged over time, and $\hat{{\bf{T}}}({\bf{x}},t)$ is variation in **T** around the mean.

As $\bar{{\bf{T}}}({\bf{x}})\,$is available for the KR from the W2 outputs, the objective is to find an RMBF for regeneration of $\hat{{\bf{T}}}({\bf{x}},t)$. As a function of space and time, $\hat{{\bf{T}}}({\bf{x}},t)$ can be decomposed into space- and time-dependent terms **Ω**(**x**) and *τ*(*t*), respectively^33^:

3 $$\hat{{\bf{T}}}({\bf{x}},t)={\sum }_{i=1}^{N}{{\boldsymbol{\Omega }}}_{{\boldsymbol{i}}}({\bf{x}}){\tau }_{i}(t)$$

For calculation of **Ω**(**x**) and *τ*(*t*), the POD approach can be used. Note that space- and time-dependent terms should be similar to the variation in **T** as much as possible. This means that the following expression must be maximized^23,34^:

4 $$\frac{1}{N}{\sum }_{i=1}^{N}\{{|({{\bf{T}}}^{i},{\bf{T}})|}^{2}/({\bf{T}},{\bf{T}})\}$$

Maximizing the above expression is equivalent to solving the following eigen-function problem:

5 $$det(1-{\bf{R}}{\bf{I}})=0$$

where **I** is the identity matrix and **R** is a Hermitian matrix calculated as^33^:

6 $${{\bf{R}}}_{i,j}=(1/N)\int {{\bf{T}}}^{(i)}({\bf{x}}){{\bf{T}}}^{(j)}({\bf{x}})d{\bf{x}}$$

The eigenvalues calculated with Eq. (5) are all positive, as shown by Eq. (7):

7 $${\lambda }_{1}\ge {\lambda }_{2}\ge \cdots \ge {\lambda }_{N}\ge 0$$

Using the eigenvalues and corresponding eigenvectors from Eq. (5), **Ω**(**x**) can be calculated as^28^:

8 $${{\boldsymbol{\Omega }}}_{1}={\sum }_{i=1}^{N}{\vartheta }_{1,i}{{\bf{T}}}^{(i)}\,,\,{{\boldsymbol{\Omega }}}_{2}={\sum }_{i=1}^{N}{\vartheta }_{2,i}{{\bf{T}}}^{(i)},\,\cdots ,\,{{\boldsymbol{\Omega }}}_{N}={\sum }_{i=1}^{N}{\vartheta }_{N,i}{{\bf{T}}}^{(i)}$$

where *ϑ*_*i*,*i*_ are eigenvectors corresponding to the eigenvalues *λ*_*i*_.

In Eq. (8), *ϑ*_*i*,*i*_ are defied as:

9 $$\begin{array}{ccc}{\vartheta }_{1,i}=[\begin{array}{c}{\vartheta }_{1,1}\\ {\vartheta }_{1,2}\\ \vdots \\ {\vartheta }_{1,N}\end{array}] & ,\,{\vartheta }_{2,i}=[\begin{array}{c}{\vartheta }_{2,1}\\ {\vartheta }_{2,2}\\ \vdots \\ {\vartheta }_{2,N}\end{array}]\,,\,\cdots ,\, & {\vartheta }_{N,i}=[\begin{array}{c}{\vartheta }_{N,1}\\ {\vartheta }_{N,2}\\ \vdots \\ {\vartheta }_{N,N}\end{array}]\end{array}$$

The time-dependent time *τ*(*t*) is calculated as^33^:

10 $${\tau }_{i}(t)=(\hat{{\bf{T}}}({\bf{x}},t),\,{{\boldsymbol{\Omega }}}_{{\boldsymbol{i}}}({\bf{x}}))$$

Both the space- and time-dependent terms can be calculated by solving an eigen-function problem. Therefore, by just using the first *K* **Ω**(**x**) and *τ*(*t*) corresponding to *K* largest eigenvalues, one can regenerate **T**(**x**,*t*)) as shown in Eq. (11). Here, *K* is much smaller than *N*.

11 $$\begin{array}{cc}{\bf{T}}({\bf{x}},t)\approx \bar{{\bf{T}}}({\bf{x}})+{\sum }_{i=1}^{N}{\tau }_{i}({\rm{t}}){{\boldsymbol{\Omega }}}_{i}({\bf{x}})\,, & K\ll N\end{array}$$

In the present study, the water temperature in the KR was simulated using Eq. (11) for a nine-year period (October 1, 2005 to September 30, 2014) and compared with that calculated by the W2 model. Root mean square error (RMSE) was used to check model performance, as shown in the subdivision “ThSSim Model Development” in Fig. S3.

The first few modes preserve almost all the energy of the system (or target variation). According to Eq. (11), the first few modes need to be used to enhance the RMBF so that it predicts the target variables in the future, an important capability of the RMBF in the field of science and engineering. However, as a result of the third principle, only the first few time-dependent components need to be estimated to construct ThSSim:

12 $$\begin{array}{cc}{\bf{T}}({\bf{x}},t+n)\approx \bar{{\bf{T}}}({\bf{x}})+{\sum }_{i=1}^{N}{\tau }_{i}(t+n){{\boldsymbol{\Omega }}}_{i}({\bf{x}})\,, & K\ll N\end{array}$$

where *τ*_*i*_(*t* + *n*) is the time-dependent term estimated in the future time *n* as described in the section “ThSSim Development Results”.

In the subdivision “ThSSim Model Development” in Fig. S3, the water temperature values simulated by W2 from October 1, 2014 to March 31, 2016 were compared with those calculated by ThSSim. In the subdivision “ThSSim Model Validation” ThSSim was validated by water temperature profiles measured during eight field sampling campaigns. The last step in the work was “ThSSim Model Application” (Fig. S3).

## Results and Discussion

### Thssim development results

**Figure 1:**
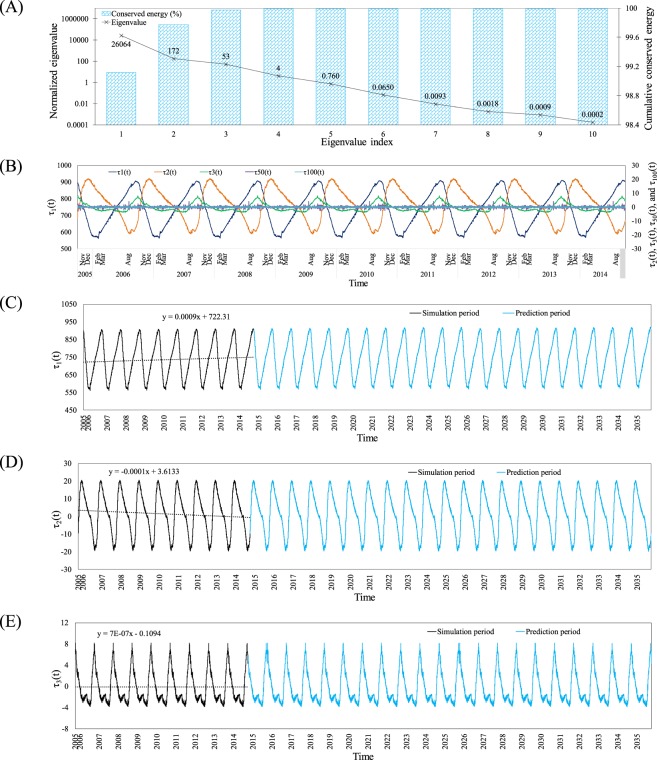
(**A**) Eigenvalues and main contribution of the first modes in cumulative conservation of the system energy, (**B**) temporal trends of time dependent terms *τ*_1_(t), *τ*_2_(t), *τ*_3_(t), *τ*_50_(t), and *τ*_100_(t) from 1^st^ October, 2005 to the end of September 2014, (**C**) The first time-dependent component extrapolated by 30^th^ September, 2035 (blue color), (**D**) The second time-dependent component extrapolated by 30^th^ September, 2035 (blue color), and (**E**) The third time-dependent component extrapolated by 30^th^ September, 2035 (blue color).

ThSSim was developed using 26,296 three-hour snapshots of water simulated by W2 over 1866 computational cells in the KR from October 1, 2005 to September 30, 2014. Results for the first 10 normalized eigenvalues calculated from positive matrix **R** with dimensions 26,296 × 26,296 are shown in Fig. 1A. The diagram shows the main contribution of the first modes in cumulative conservation of the system's energy (water temperature variance in the KR), where this value reaches about 99.97% for the first three modes.

**Figure 2:**
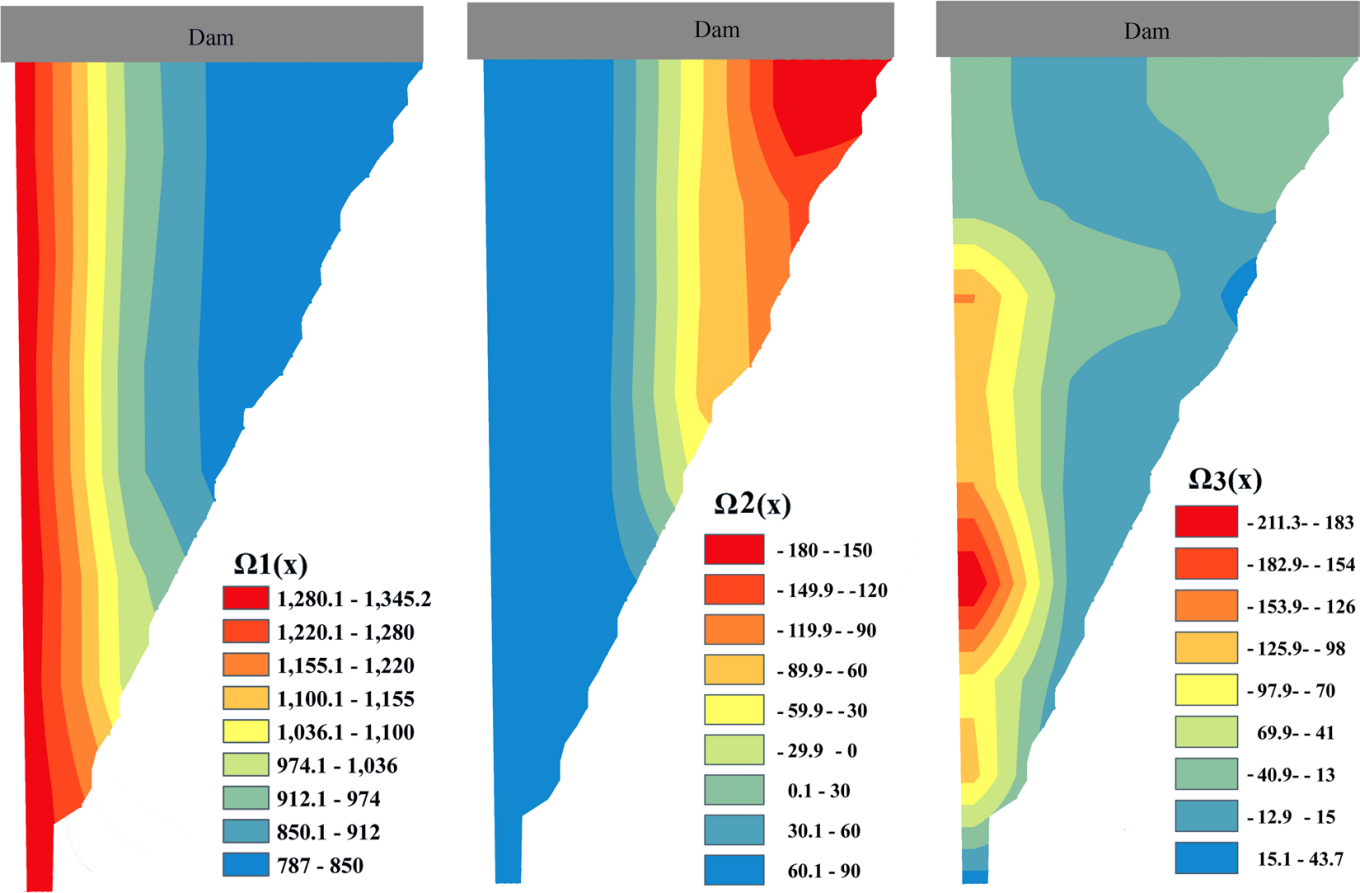
The first to third space-dependent terms (**Ω**_1_(**x**), **Ω**_2_(**x**), and **Ω**_3_(**x**) respectively).

The space-dependent terms **Ω**_1_(**x**) to **Ω**_3_(**x**) are depicted in Fig. 2. **Ω**_1_(**x**), corresponding to the first eigenvalue, represents the dominant mode of the spatial variation in water temperature in the KR. A decreasing trend in **Ω**_1_(**x**) values with depth can be seen in the reservoir. This means high variation in water temperature in the surface layers, which are highly affected by atmospheric driving forces. Meanwhile, the deeper layers in the KR are isolated from the atmospheric driving forces in the almost duration of year and just experience a brief mixing during few months in winter^31^. Additionally, heat transport by molecular diffusion to the bottom layers is so slow that needs about a month to penetrate about 1 m in depth of such stratified reservoir^5^. Therefore, during ThS the deeper layers in the KR are isolated, so that they experience lower variations of water temperature compared to the surface layers, as shown in Fig. 2. Also, field measurements showed the water temperature values reduced in depth so that in the bottom it reached about a half of those observed in the surface water^31^. This fact has properly reflected in the first mode as well, where the magnitude of first mode in the surface water is about two times greater than those shown in the bottom layers. Thus, it can be concluded that **Ω**_1_(**x**) accurately depicts the spatial distribution of ThS in the KR. Contrary to **Ω**_1_(**x**), **Ω**_2_(**x**) shows an increasing trend in depth, so that the deeper layers experience more variation than surface layers. From a physical point of view, this situation represents a mixing process in the KR when the thermal gradient becomes so low between surface and bottom layers that an overturn occurs. In general, the both **Ω**_1_(**x**) and **Ω**_2_(**x**) are quasi one-dimensional in structure, pointing that a one-dimensional model would be enough to capture them. Calculated **Ω**_3_(**x**) in the KR shows a roughly decreasing trend in depth. This may represent thermal stability in the KR, as with **Ω**_1_(**x**), but the trend is not as regular as that in **Ω**_1_(**x**), especially in deep locations close to the dam structure. Therefore, this mode shows the sense of using a two-dimensional model such as W2. However, due to very small contribution of the second and third modes to conservation of the system's energy, less than 5% (see Fig. 1A), they might be less important in practical application.

Time-dependent terms *τ*_1_(t), *τ*_2_(t), *τ*_3_(t), *τ*_50_(t), and *τ*_100_(t) are illustrated in Fig. 1B, where *τ*_1_(t), corresponding to the first eigenvalue, is the most important. It is noteworthy that the time-dependent terms corresponding to higher eigenvalue indices, e.g., *τ*_50_(t) and *τ*_100_(t), are so small that they just fluctuate around zero. Therefore, Fig. 1B shows the main contribution of the first few time-dependent terms compared with others. More specifically, *τ*_1_(t) represents the time variation in water temperature in the KR, which shows an increasing trend starting from early March and continuing to early October each year. Thereafter, this term follows a decreasing trend from early October to early March each year. These periods of the year are characterized by increasing and decreasing air temperature, respectively, in the study area. Therefore, *τ*_1_(t) accurately represents the variation in atmospheric driving forces and consequently the variation in water temperature in the KR. Contrary to *τ*_1_(t), *τ*_2_(t) shows roughly decreasing and increasing trends during warm (spring, fall, and summer) and cold (winter) seasons, respectively, in each year. From a physical point of view, this situation may contribute to the variation in water temperature in the bottom layers, where a decreasing and increasing trend is observed in the KR during warm and cold seasons, respectively. This is discussed further in section 4.3 of this paper. Similar to *τ*_1_(t) and *τ*_2_(t), *τ*_3_(t) fluctuates each year, but comparing the magnitude of this time-dependent term with that of *τ*_1_(t) indicates a very small contribution of *τ*_3_(t) to conservation of the system's energy. In general, the time-dependent terms *τ*_1_(t) of ThSSim represent all time-dependent variables/factors in the system responses, i.e. hydro-meteorological and reservoir operation strategies during the simulation period^31^.

**Figure 3:**
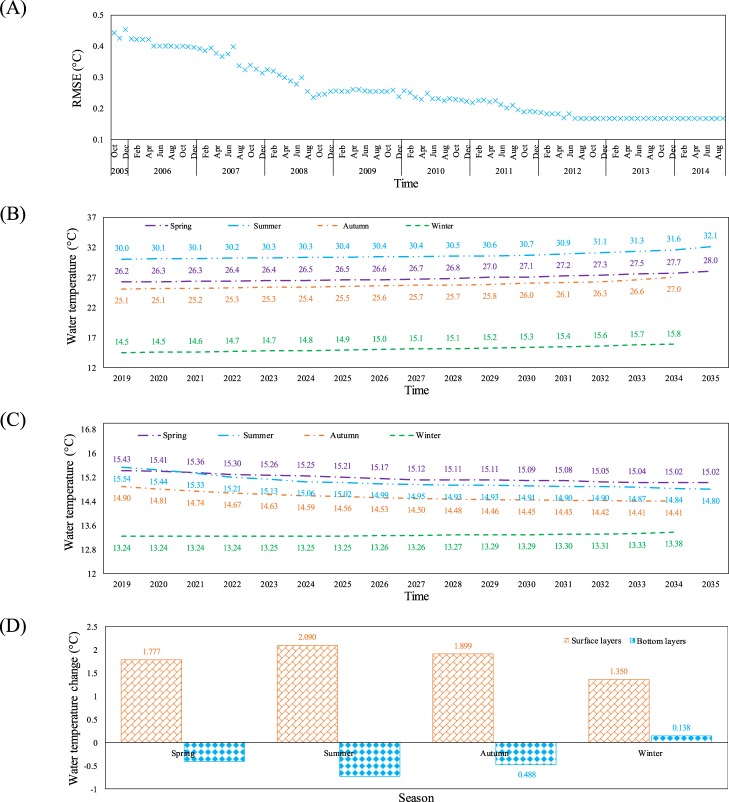
(**A**) The series of monthly errors (RMSE) between the water temperature simulated by W2 and ThSSim in the KR from 1^st^ October, 2005 to the end of September 2014, (**B**) Trends of mean water temperature in the surface layers of KR for each season during 2019–2035, (**C**) Trends of mean water temperature in the bottom layers of KR for each season during 2019–2035, and (**D**) Difference between mean water temperature in the surface and bottom layers of KR for each season during 2019–2035.

Considering the above, the first few modes contribute almost all the system's energy. This means that using additional modes with too small a contribution in the system's energy for development of the ThSSim just results in model complexity, rather than increasing its accuracy. In this regard, values greater than 99% of the system's energy have been used by different authors^25,33^. In this study, the first three modes, which represent around 99.97% of the system's energy, were selected to develop ThSSim. A comparison was then carried out between the water temperature in the KR simulated by W2 and ThSSim during the period October 1, 2005 to September 30, 2014. The series of monthly errors (RMSE) between the water temperature simulated by W2 and ThSSim in the KR revealed no a distinct difference between W2 and ThSSim (Fig. 3A). The maximum RMSE value, 0.54 °C, was found for the early simulation period and RSME reached a constant value of around 0.16 °C at the end of simulation period (September 30, 2014).

To add predictive ability to ThSSim, we extrapolated the first three time-dependent components to September 30, 2035, as shown in Figs. 1C–E, respectively. The extrapolation method used was simple and straightforward based on general increasing/decreasing linear trends of the time-dependent terms as shown in Figs. 1C–E. For example, as Fig. 1C shows, *τ*_1_(t) increased with a slow gradient ascent (equal to 0.0009) from October 1, 2005 to September 30, 2014. By considering a gradient ascent equal to 0.0009, we shifted the cyclic trend of *τ*_1_(t) observed from October 1, 2005 to September 30, 2014 to the future times, i.e. October 1, 2015 to September 30, 2035 (Fig. 1C). The same procedure was used to extrapolate *τ*_2_(t) and *τ*_3_(t) in the future as shown in Fig. 1D,E, respectively. Note that *τ*_1_(t), which represents the trend in the time-dependent term for water temperature variation in the KR, clearly reveals warming in the reservoir during the nine years. This finding is in line with a reported increasing trend in air temperature in the study area during the period^35^. In addition, very slow decreasing and increasing trends in *τ*_2_(t) and *τ*_3_(t) can be seen in Fig. 1D,E, respectively, while *τ*_2_(t) and *τ*_3_(t) extrapolated from October 1, 2014 to September 30, 2035 are shown by blue color in the respective diagrams.

**Figure 4:**
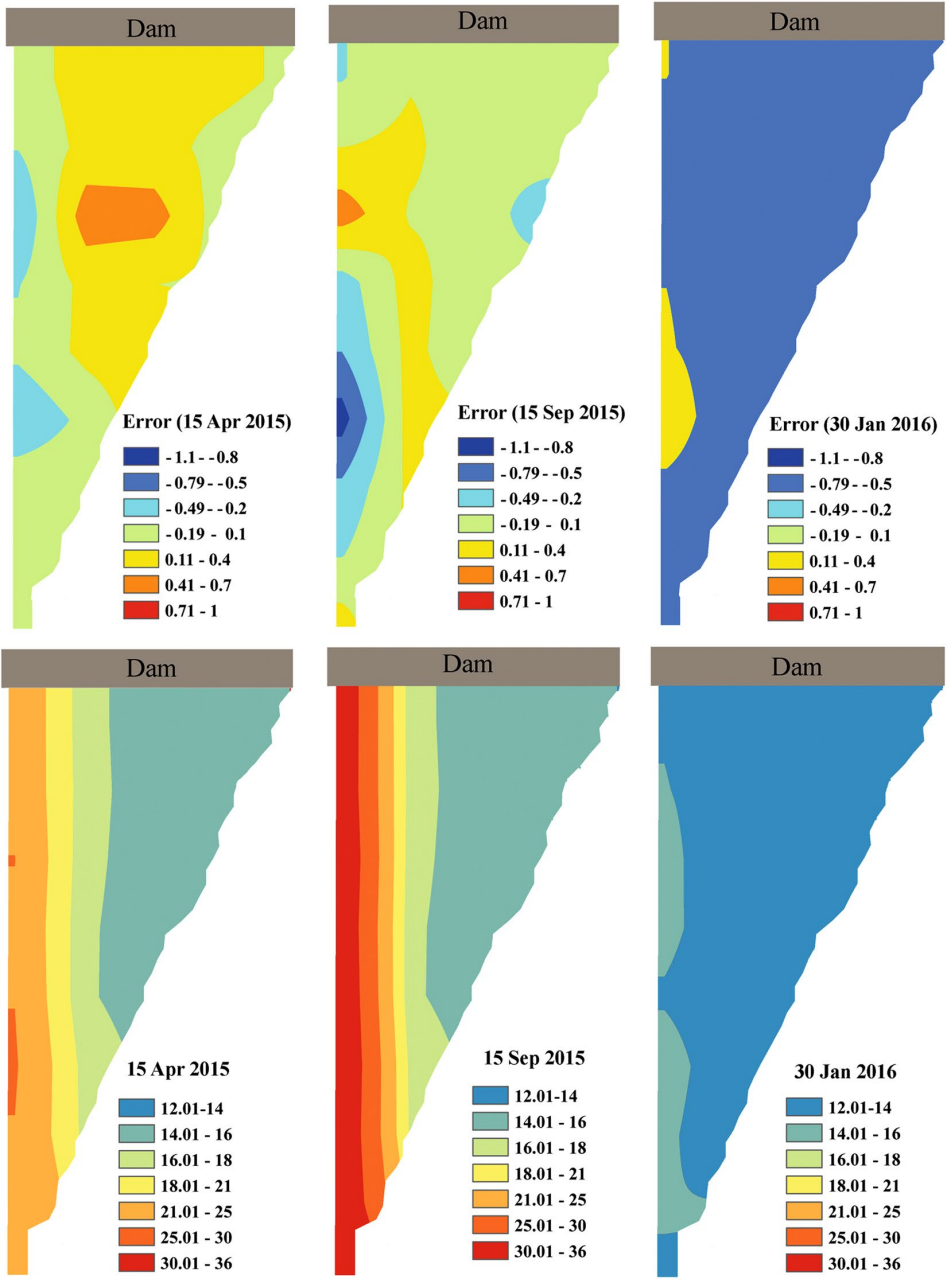
Spatial distribution of water temperature simulated by ThSSim and difference between this model and W2 in the KR for selective days 15^th^ April and September, 2015 and 30^th^ January, 2016.

Having **Ω**_1_(**x**) to **Ω**_3_(**x**) and also using future values for *τ*_1_(t) and *τ*_3_(t), ThSSim was used to predict water temperature in the KR from October 1, 2014 to March 31, 2016. The results simulated by ThSSim were then compared with those obtained using the W2 model. The RMSE between ThSSim and W2 results was about 0.52 °C which is similar as the maximum error in the calibration as a shown in Fig. 3A. This may result from extrapolation of time-dependent terms in the future (after 2014 until 2035) that usually brings some errors in practical application of ThSSim. More specifically, Fig. 4 shows the spatial distribution of KR water temperature simulated by ThSSim and the difference between this and that obtained using W2 for three selected days: April 15 and September 15, 2015 and January 30, 2016. As can be seen, the ThSSim results matched well those obtained by W2, with the calculated error ranging between −0.65 and 0.65 for April 15, 2015, between −1.2 and 1.2 for September 15, 2015, and between −0.29 and 0.29 for January 30, 2016.

### Thssim validation results

**Figure 5:**
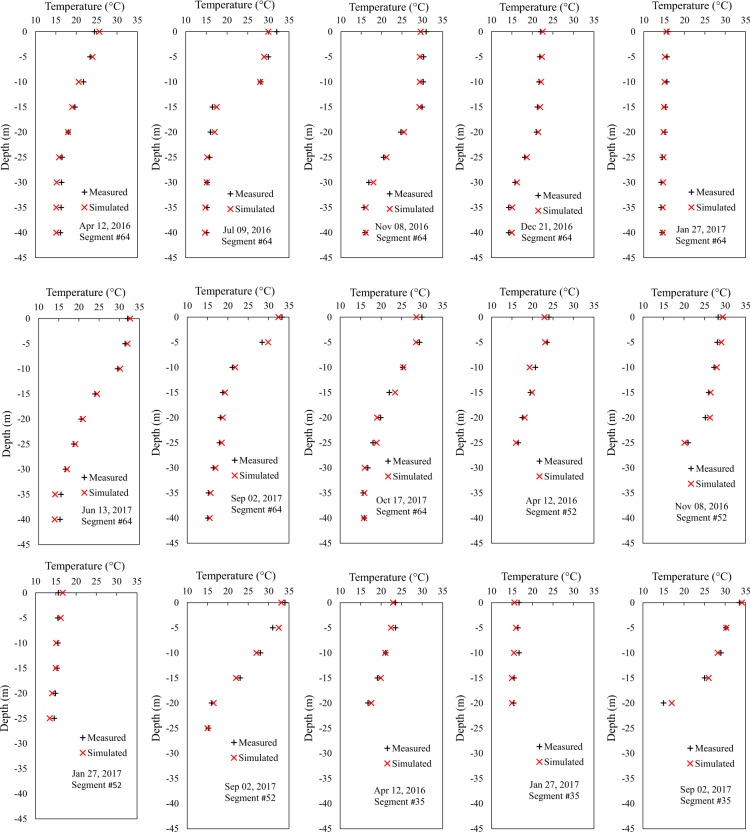
Water temperature profiles measured at segments 64, 52, and 35 in the KR vs those simulated by ThSSim model for all sampling campaigns.

ThSSim model performance was evaluated by comparing the water temperature profiles measured at segments 64 (adjacent to the dam structure), 52 (13 km far from the dam structure), and 35 (29 km far from the dam structure) in the KR with those simulated by the model (Fig. 5). There was good agreement between the water temperature simulated by ThSSim and that measured in all sampling campaigns. From a numerical point of view, mean absolute error values between the results simulated by ThSSim and the measured values were 0.68, 0.73, and 0.77 °C at segments 64, 52, and 35, respectively. These values clearly demonstrate excellent performance of ThSSim in prediction of water temperature in the KR. In addition, given 2D model spatial structure and good performance of the model in three different sampling points alongside the KR, simulated horizontal heterogeneity using the ThSSim were also verified.

### Future prediction (2019–2035)

After successful development and validation of ThSSim, future prediction of water temperature in the KR was performed (Figs. 3B–D). Here, surface layers refer to the first to third layers, located between depths 0 to 3 m in the KR. Warming trends in mean surface water temperature for four seasons (spring to winter) are shown in Fig. 3B during the simulation period 2019–2035. The warming trend was at a maximum for summer season as simply shown in Fig. 3D, where mean water temperature increased from 30.0 °C in 2019 to 32.1 °C in 2035 (Fig. 3B). This warming value in surface water temperature in a 15-year period is considerable. Since variation in surface water temperature in the reservoir is highly affected by climate indices, this trend should be a result of severe decreasing and increasing trends in precipitation and air temperature, respectively, in the study area. This assumption is supported by previous findings indicating that the length of the dry period in the study area will increase by up to 25% in the period 2020–2040^36^. Climate models also predict a warming trend in the future over the KRW^37^. Mean water temperature in surface layers showed a smaller positive trend for winter season as well (Fig. 3B), where the difference during the simulation period was about 1.3 °C as simply shown in Fig. 3D. This finding matches well the future air temperature variation in the KRW predicted by Solaymani and Gosain^38^, who concluded that increasing ambient air temperature in the KRW will be smaller in winter than in summer. As Fig. 3D shows, warming in mean temperature in surface layers in spring and autumn was 1.8 and 1.9 °C, respectively.

A similar analysis as for surface layers was performed to calculate the trends in mean water temperature in the bottom layers of the KR for each season 2019–2035 (Fig. 3C). Mean water temperature in bottom layers showed a cooling trend for three seasons (spring, summer, and autumn) from 2019 to 2035, possibly due to the longer isolation period of bottom layers in the KR in the simulation period, as discussed in Section 4.4. Therefore, a cooling trends for these seasons, when the KR is thermally stable, can be expected. For winter season, the results show a positive trend with a small vertical gradient in the KR in the future (Fig. 3C). This situation is accurately reflected by *τ*_2_(t) as it shows roughly decreasing and increasing trends during warm (spring, fall, and summer) and cold (winter) seasons, respectively, in each year.

**Figure 6:**
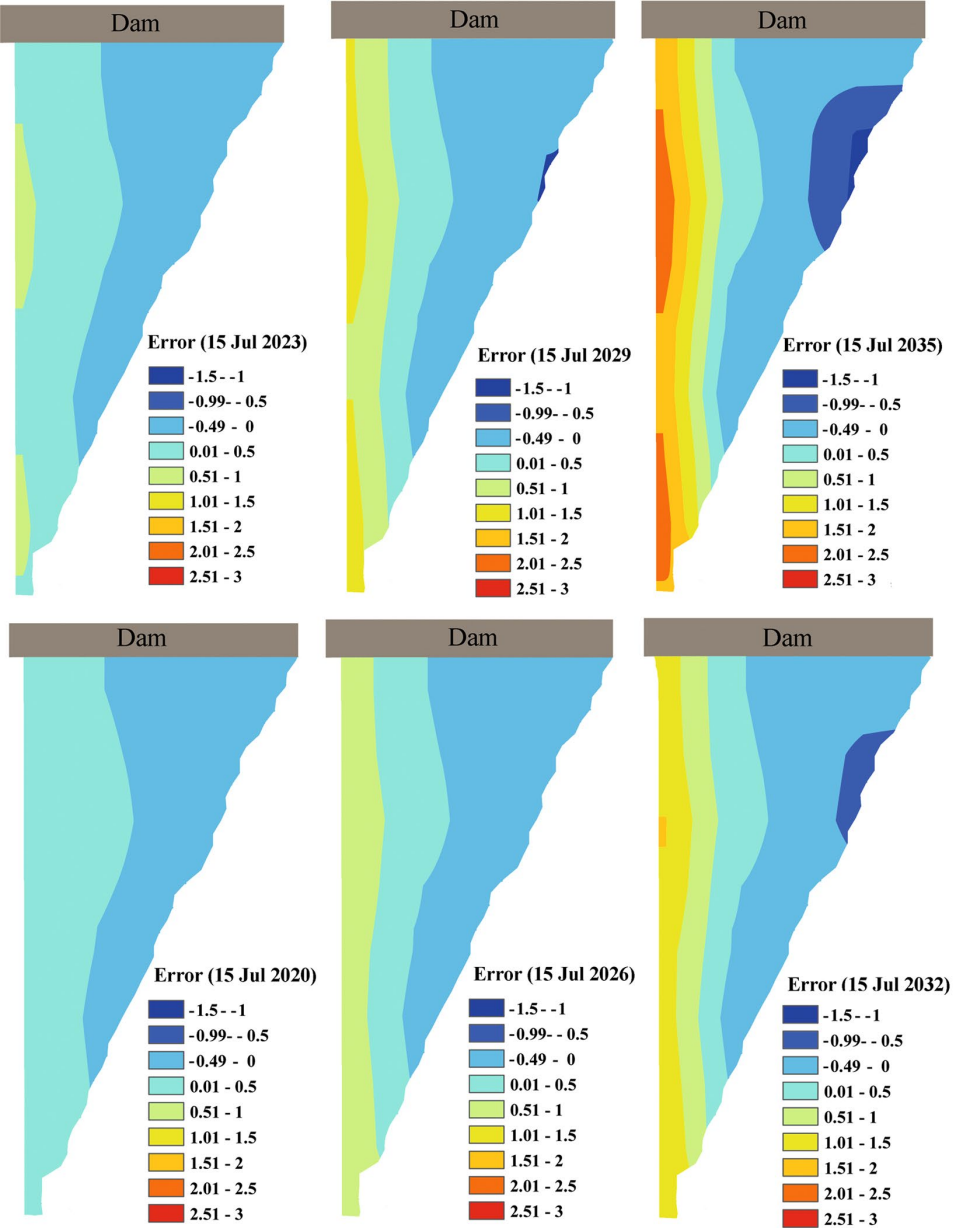
The error spatially integrated in the KR resulting from differences between water temperature simulated by ThSSim in the KR on a specific Julian day (July 15) in 2018 and that Julian day in the future years 2020, 2023, 2026, 2029, 2032, and 2035.

Figure 6 shows the error spatially integrated in the KR resulting from differences between water temperature simulated by ThSSim in the KR on a specific Julian day (July 15) in 2018 and that Julian day in the future years 2020, 2023, 2026, 2029, 2032, and 2035. These diagrams clearly indicate a warming and cooling in surface and bottom layers of the KR, respectively, from 2019 to 2035.

### Beginning and end times of the ThS

**Figure 7:**
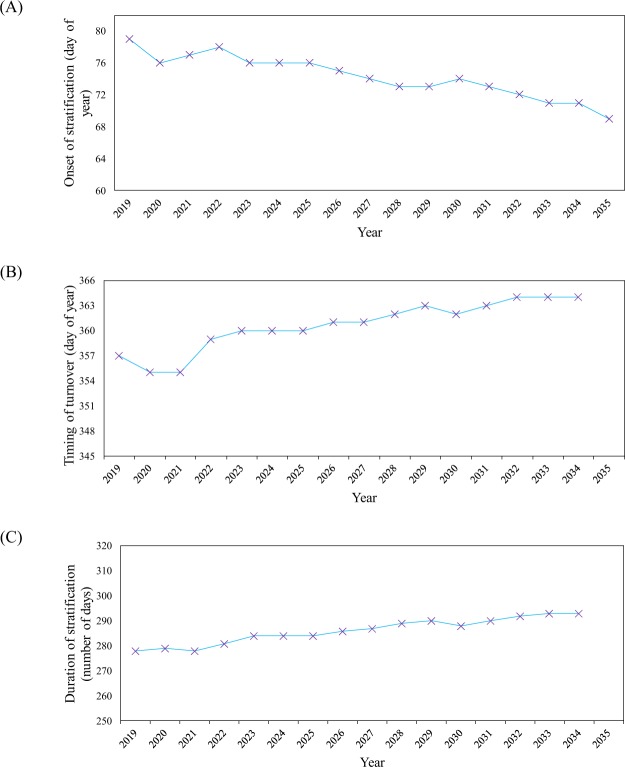
The results for (**A**) beginning times of ThS in the KR during 2019–2035, (**B**) ending times of ThS in the KR during 2019–2035, and (**C**) during of ThS in the KR for period 2019–2035.

Having water temperature profiles simulated by ThSSim in segment 64, the time of beginning and end of ThS was calculated annually for the KR. In this regard, threshold values greater than 1 °C between two adjacent 1-m intervals were selected to determine thermal stability in the water column, as suggested by Stainsby *et al*.^39^ and Hadley *et al*.^40^. The results for beginning and end times and for duration of ThS in the KR are given in Figs. 7A,C, respectively. As can be seen in Fig. 7A, the onset of ThS shows a decreasing trend, so that it starts on Julian day 79 in the year 2019 and declines to Julian day 69 in the year 2035. An increasing trend in the breakdown time of ThS is clearly shown in Fig. 7B. This means that onset of mixing in the KR will be delayed in the future, so that it shifts from Julian day 357 in 2019 to 364 in 2034. These decreasing and increasing trends for onset and breakdown of ThS, respectively, will increase the duration of thermal stability in the KR. Therefore, the results simulated by ThSSim indicate that the duration of ThS will increase from 278 days in 2019 to 293 days in 2034 (15-day increase over the 15-year period), as shown in Fig. 7C. This increasing trend in duration may be a result of changes in atmospheric driving forces, especially episodic wind events, as reported by Coates *et al*.^37^, Golosov *et al*.^41^, Butcher *et al*.^42^, Edlund *et al*.^43^, Ladwig *et al*.^44^, and Mi *et al*.^45^. Jamali *et al*.^46^, Kamali *et al*.^47^, and Fereidoon and Koch^48^ presented similar results on changes in the climatological driving forces in southern parts of the KRW. Therefore, it can be concluded that in future, atmospheric driving forces will contribute to longer periods of ThS in the KR. On the other hand, the reservoir is a deep water body, which makes it difficult to achieve complete mixing^30^. Therefore, the reservoir may face problems deriving from longer thermal stability in the future, such as diffusion of some chemical compounds from the bottom sediments.

## Conclusions

Simulation of reservoir thermal stratification using numerical models can provide the required information for managers to plan the best solution for improving reservoir water quality. However, numerical models are based on complex formulation, so that they can simultaneously consider the effect of all driving forces on ThS, and produce large volumes of outputs, which may need to be post-processed by a separate program to capture the dominant ThS modes during the simulation period/scenario.

In this study, a simple tool entitled ThSSim was developed to address these issues in modeling ThS processes. The tool, which is based on linked W2 and POD models, but enhanced so that it can predict ThS in the future, was applied to the Karkheh Reservoir in Iran as a case study. The results indicated good performance of ThSSim, as water temperature profiles simulated by this tool matched well those obtained using the W2 model. Practical application of ThSSim showed warming and cooling trends in surface and bottom layers, respectively, in the KR during 2019–2035. These findings are consistent with climate predictions for the study area.

However, it should be noted that the time-dependent terms of ThSSim change in the time as learned from the patterns embedded in the historical data computed by W2 model (2005 to 2016). These patterns are a result of the time-dependent variables/factors in the system responses, i.e. hydro-meteorological and reservoir operation strategies during the simulation period. A simple extrapolation method was used to modify these patterns expressed in time dependent terms by 2035, as well. By considering these explanations, the future trends in temperature stratification got would happen if the extrapolation method works well during 2019–2035.

## Supplementary information

Supplementary Information for
