# Supplementary material for: ThSSim: A novel tool for simulation of reservoir thermal stratification
Source: Sci Rep. 2019 Dec 6;9:18524. doi: 10.1038/s41598-019-54433-2 (PMC6898630; doi:10.1038/s41598-019-54433-2)
Supplement: Supplementary file 1 — Supplementary Information for [file 41598_2019_54433_MOESM1_ESM.docx]

Supplementary Information for:

ThSSim: A novel tool for simulation of reservoir thermal stratification

Roohollah Noori^1,2^, Fuqiang Tian^2^, Guangheng Ni^2^, Rabin Bhattarai^3^, Farhad Hooshyaripor^4^, Bjön Klöve^5^

^1^School of Environment, College of Engineering, University of Tehran, Tehran, 1417853111, Iran

^2^Department of Hydraulic Engineering, State Key Laboratory of Hydroscience and Engineering, Tsinghua University, Beijing, 100084, China

^3^Department of Agricultural and Biological Engineering, University of Illinois at Urbana Champaign, 1304 W Pennsylvania Ave, Urbana IL, 61801, USA

^4^Department of Civil Engineering, Science and Research Branch, Islamic Azad University, Tehran, 1477893855, Iran

^5^Water Resources and Environmental Engineering Research Unit, Faculty of Technology, PO Box 4300, 90014 University of Oulu, Finland

^*^Correspondence: Roohollah Noori

Department of Environmental Engineering, School of Environment, College of Engineering,

University of Tehran

Enqelab Avenue, Qods Street, Azin Alley, P.O. Box: 14155-6135, Tehran, Iran

Tel: +982161113584; Fax: +982166407719; E-mail: [noor@ut.ac.ir](mailto:noor@ut.ac.ir)

ORCID: <http://orcid.org/0000-0002-7463-8563>

**SI Figure Captions**

**Figure S1.** Location of the KRW in Iran

**Figure S2.** Network of dam reservoirs designed to protect the people from floods and provide water for different usages in the KRW

**Figure S3.** Details of the present study methodology


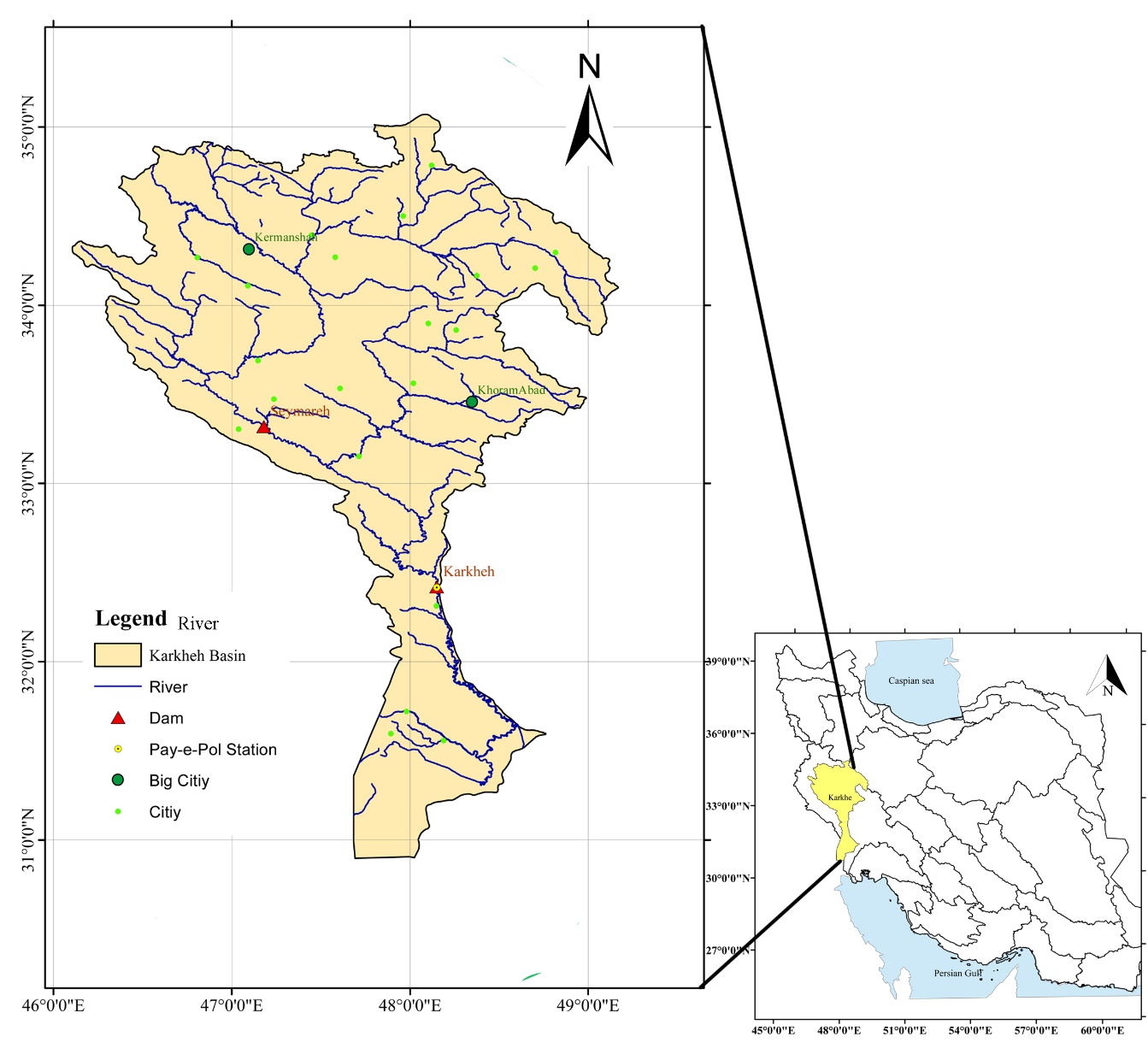


**Figure S1**


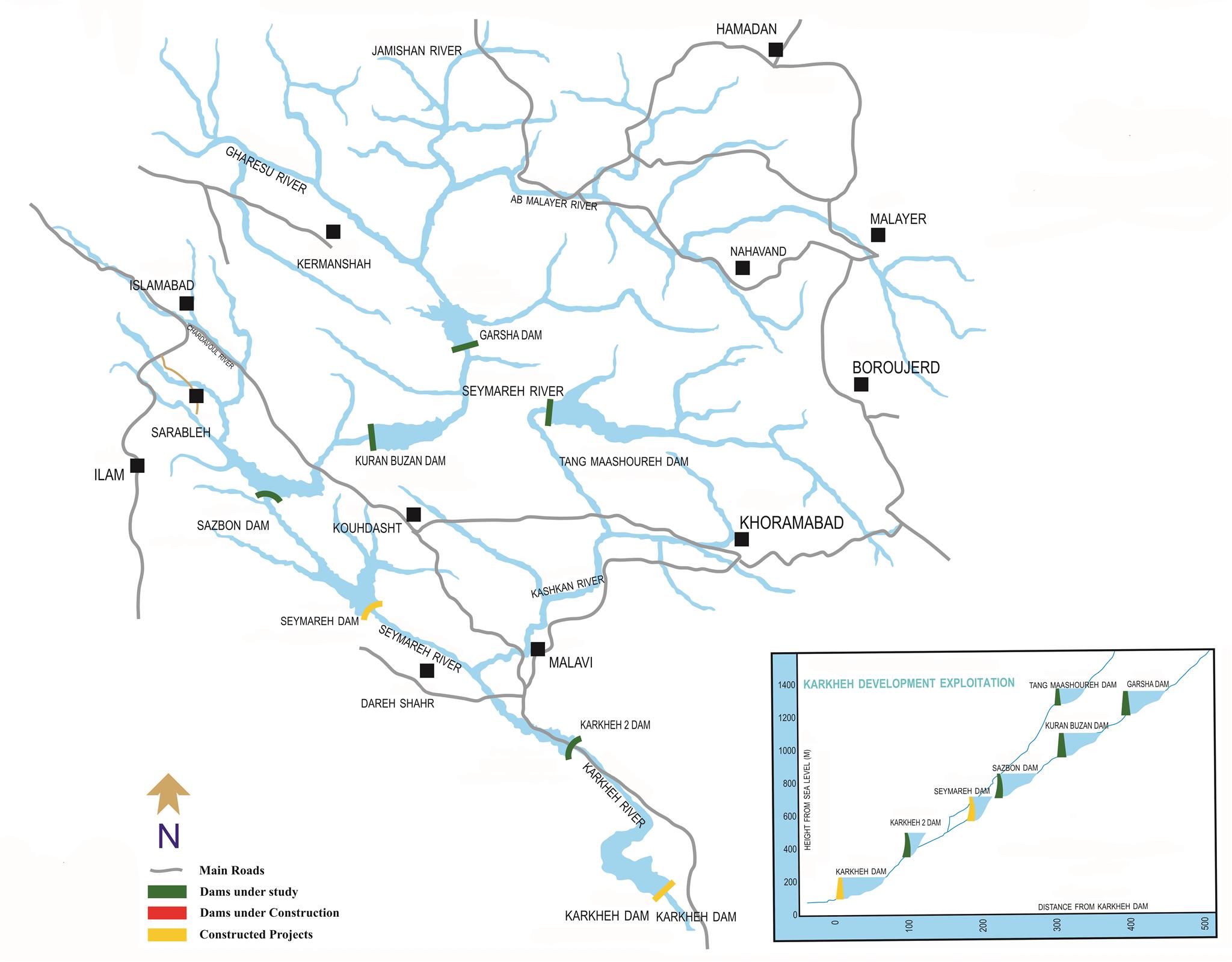


**Figure S2**


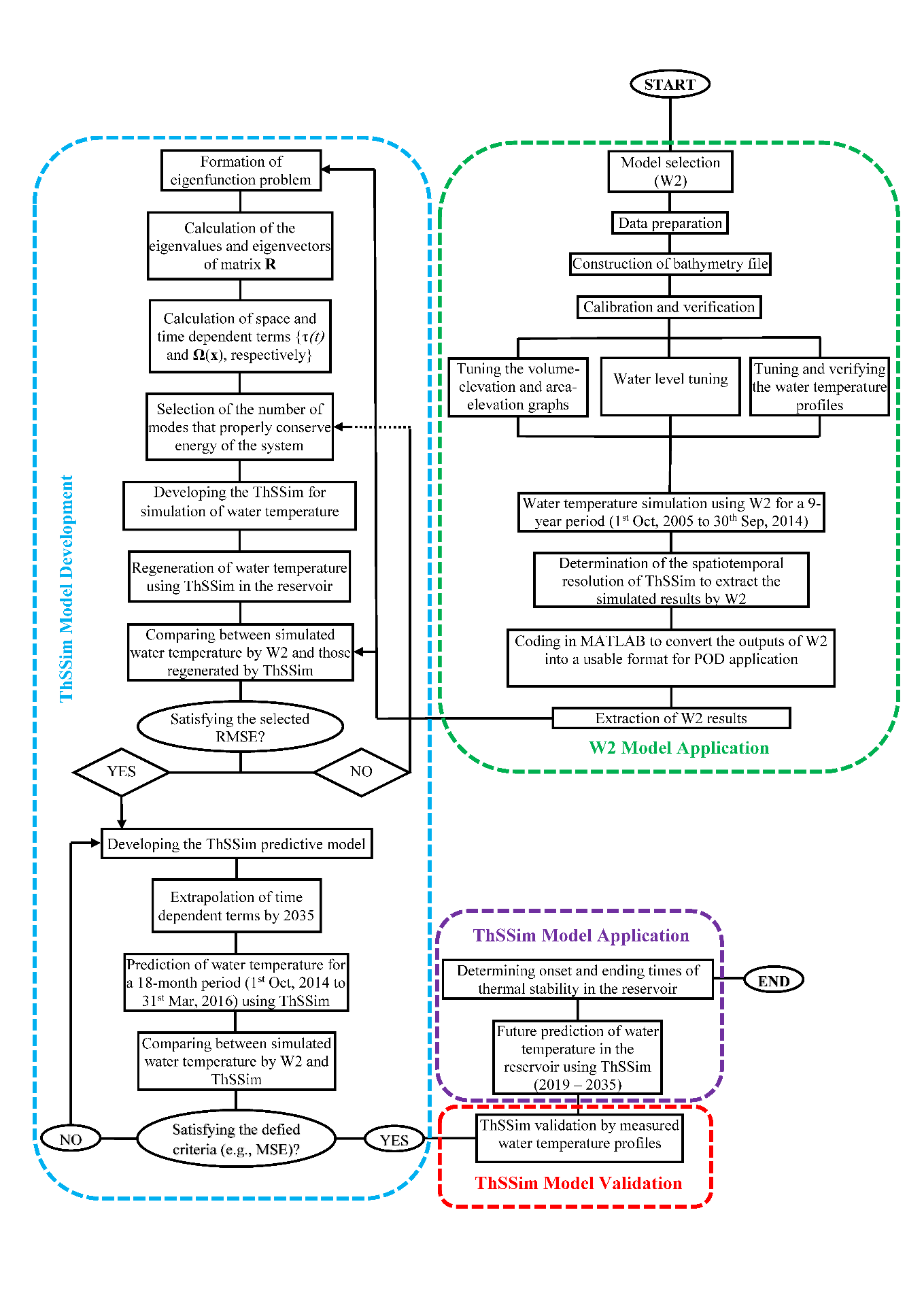


**Figure S3**
